# PrImary decompressive Craniectomy in AneurySmal Subarachnoid hemOrrhage (PICASSO) trial: study protocol for a randomized controlled trial

**DOI:** 10.1186/s13063-022-06969-4

**Published:** 2022-12-20

**Authors:** Erdem Güresir, Tim Lampmann, Simon Brandecker, Marcus Czabanka, Rolf Fimmers, Jens Gempt, Patrick Haas, Amer Haj, Ramazan Jabbarli, Darius Kalasauskas, Ralph König, Dorothee Mielke, Robert Németh, Marvin Darkwah Oppong, Andrej Pala, Vincent Prinz, Florian Ringel, Constantin Roder, Veit Rohde, Karl-Michael Schebesch, Arthur Wagner, Christoph Coch, Hartmut Vatter

**Affiliations:** 1 Department of Neurosurgery, University Hospital Bonn, Venusberg-Campus 1, D-53127 Bonn, Germany; 2 Department of Neurosurgery, Johann Wolfgang Goethe-University of Frankfurt, Schleusenweg 2-16, D-60529 Frankfurt, Germany; 3 Institute of Medical Biometry, Informatics and Epidemiology, University Hospital Bonn, Venusberg-Campus 1, D-53127 Bonn, Germany; 4 Department of Neurosurgery, Klinikum rechts der Isar, School of Medicine, Technical University Munich, Ismaninger Str. 22, D-81675 Munich, Germany; 5 Department of Neurosurgery, Eberhard Karls University Tübingen, Hoppe-Seyler-Str. 3, D-72076 Tübingen, Germany; 6 Department of Neurosurgery, University Medical Center Regensburg, Franz-Josef-Strauss Allee 11, D-93053 Regensburg, Germany; 7 Department of Neurosurgery and Spine Surgery, University Hospital of Essen, Hufelandstraße 55, D-45147 Essen, Germany; 8 Department of Neurosurgery, Mainz University Hospital, Langenbeckstraße 1, D-55131 Mainz, Germany; 9 Department of Neurosurgery, University of Ulm/BKH Günzburg, Lindenallee 2, D-89312 Günzburg, Germany; 10 Department of Neurosurgery, Georg-August-University Göttingen, Robert-Koch-Straße 40, D-37075 Göttingen, Germany; 11 Clinical Study Core Unit, Study Center Bonn (SZB), University Hospital Bonn, Venusberg-Campus 1, D-53127 Bonn, Germany

**Keywords:** Early brain injury, Intracranial pressure, Decompressive craniectomy, Subarachnoid hemorrhage, Intracranial aneurysm, Randomized trial

## Abstract

**Background:**

Poor-grade aneurysmal subarachnoid hemorrhage (SAH) is associated with poor neurological outcome and high mortality. A major factor influencing morbidity and mortality is brain swelling in the acute phase. Decompressive craniectomy (DC) is currently used as an option in order to reduce intractably elevated intracranial pressure (ICP). However, execution and optimal timing of DC remain unclear.

**Methods:**

PICASSO resembles a multicentric, prospective, 1:1 randomized standard treatment-controlled trial which analyzes whether primary DC (pDC) performed within 24 h combined with the best medical treatment in patients with poor-grade SAH reduces mortality and severe disability in comparison to best medical treatment alone and secondary craniectomy as ultima ratio therapy for elevated ICP. Consecutive patients presenting with poor-grade SAH, defined as grade 4–5 according to the World Federation of Neurosurgical Societies (WFNS), will be screened for eligibility. Two hundred sixteen patients will be randomized to receive either pDC additional to best medical treatment or best medical treatment alone. The primary outcome is the clinical outcome according to the modified Rankin Scale (mRS) at 12 months, which is dichotomized to favorable (mRS 0–4) and unfavorable (mRS 5–6). Secondary outcomes include morbidity and mortality, time to death, length of intensive care unit (ICU) stay and hospital stay, quality of life, rate of secondary DC due to intractably elevated ICP, effect of size of DC on outcome, use of duraplasty, and complications of DC.

**Discussion:**

This multicenter trial aims to generate the first confirmatory data in a controlled randomized fashion that pDC improves the outcome in a clinically relevant endpoint in poor-grade SAH patients.

**Trial registration:**

DRKS DRKS00017650. Registered on 09 June 2019.

**Supplementary Information:**

The online version contains supplementary material available at 10.1186/s13063-022-06969-4.

## Administrative information

Note: the numbers in curly brackets in this protocol refer to SPIRIT checklist item numbers. The order of the items has been modified to group similar items (see http://www.equator-network.org/reporting-guidelines/spirit-2013-statement-defining-standard-protocol-items-for-clinical-trials/).

**Table:** 

Title {1}	**P**r**I**mary decompressive **C**raniectomy in **A**neury**S**mal **S**ubarachnoid hem**O**rrhage (**PICASSO**) trial: study protocol for a randomized controlled trial.
Trial registration {2a and 2b}.	DRKS-ID: DRKS00017650
Protocol version {3}	19. July 2019, protocol version 2.0
Funding {4}	Medical Faculty of the University of Bonn via the “Förderinstrument Klinische Studien der Kommission für Klinische Studien”
Author details {5a}	^1^Department of Neurosurgery, University Hospital Bonn, Venusberg-Campus 1, D-53127 Bonn, Germany ^2^Department of Neurosurgery, Johann Wolfgang Goethe-University of Frankfurt, Schleusenweg 2-16, D-60529 Frankfurt, Germany ^3^Institute of Medical Biometry, Informatics and Epidemiology, University Hospital Bonn, Venusberg-Campus 1, D-53127 Bonn, Germany ^4^Department of Neurosurgery, Klinikum rechts der Isar, School of Medicine, Technical University Munich, Ismaninger Str. 22, D-81675 Munich, Germany ^5^Department of Neurosurgery, Eberhard Karls University Tübingen, Hoppe-Seyler-Str. 3, D-72076, Tübingen, Germany ^6^Department of Neurosurgery, University Medical Center Regensburg, Franz-Josef-Strauss Allee 11, D-93053 Regensburg, Germany ^7^Department of Neurosurgery and Spine Surgery, University Hospital of Essen, Hufelandstraße 55, D-45147 Essen, Germany ^8^Department of Neurosurgery, Mainz University Hospital, Langenbeckstraße 1, D-55131, Mainz, Germany ^9^Department of Neurosurgery, University of Ulm/BKH Günzburg, Lindenallee 2, D-89312 Günzburg, Germany ^10^Department of Neurosurgery, Georg-August-University Göttingen, Robert-Koch-Straße 40, D-37075 Göttingen, Germany ^11^Clinical Study Core Unit, Study Center Bonn (SZB), University Hospital Bonn, Venusberg-Campus 1, D-53127 Bonn, Germany
Name and contact information for the trial sponsor {5b}	Investigator initiated trial. Principal Investigator: Prof. Dr. med. Erdem Güresir Department of Neurosurgery, University Hospital Bonn, Venusberg-Campus 1, D-53127 Bonn, Germany
Role of sponsor {5c}	This is an investigator initiated trial. The funder had no input in the design, conduct or future data analysis and interpretation of the study.

## Introduction

### Background and rationale {6a}

Poor-grade aneurysmal subarachnoid hemorrhage (SAH), defined as grades 4 and 5 according to the World Federation of Neurosurgical Societies (WFNS) [1], is associated with poor neurological outcome and high mortality [2–5].

The rupture of an intracranial aneurysm can cause brain swelling primarily due to severe damage of brain tissue and secondarily due to vasospasm followed by infarction that both leads to elevation of intracranial pressure (ICP). Studies have shown that brain swelling and elevated ICP are known to worsen outcomes following SAH [6–8]. Despite this, significant predictors of unfavorable outcomes in patients with poor-grade SAH are patient age, WFNS grade 5, signs of cerebral herniation, aneurysm size, and space-occupying hematoma [9].

Decompressive craniectomy (DC) is a well-established surgical intervention aiming to reduce increased ICP. After traumatic brain injury (TBI) and space-occupying stroke, DC has been shown to successfully reduce elevated ICP and also improve patient’s outcome in those strokes [10–13].

Besides a proven lifesaving effect in general, it is still debatable if an early decompression within 24 h after SAH—i.e., primary DC (pDC)—provides a better long-term functional outcome with lower rates of severe disability and dependency rates of patients or leads to a higher number of survival with dependent patients. A retrospective series of patients treated with a pDC in poor-grade SAH indicates that a pDC may be warranted in this severely ill subset of patients [14]. However, despite these promising data, there are no data from randomized controlled trials proving or disproving the beneficial effect of pDC, a rather aggressive surgical treatment compared to a neuro-intensive medical therapy with a secondary DC only performed if ICP cannot be handled by other treatment options [15–34].

Thus, we aim to investigate the effect of pDC in a prospective randomized trial regarding the mortality rate and the degree of disability of these patients.

### Objectives {7}

Comparison of the combination of mortality and severe disability between study arms, assessed by the modified Rankin Scale [35] (mRS) between both study arms 12 months after the subarachnoid hemorrhage, dichotomized in “favourable” (mRS 0–4) and “unfavourable” (mRS 5–6) outcome.

### Trial design {8}

**Fig. 1:**
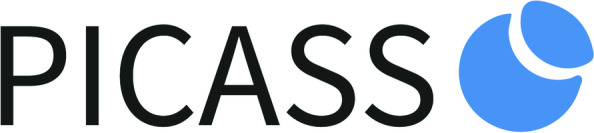
Trial logo

PICASSO resembles a multicentric, prospective, 1:1 randomized, standard treatment-controlled, superiority study with blinded ratings with regard to the mRS assessment during telephone interviews. It will follow the recommendation and suggestion of CONSORT 2010 (Consolidated Standards of Reporting Trials). All patients included in the study will initially receive standard treatment according to the institutional guidelines of each participating site [4]. In the experimental arm, patients will undergo a pDC of at least 12 cm within the first 24 h after ictus. In the control arm, patients may undergo a secondary DC as ultima ratio therapy for therapy-refractory increased intracranial pressure (Fig. 1).

## Methods: participants, interventions, and outcomes

### Study setting {9}

**Fig. 2:**
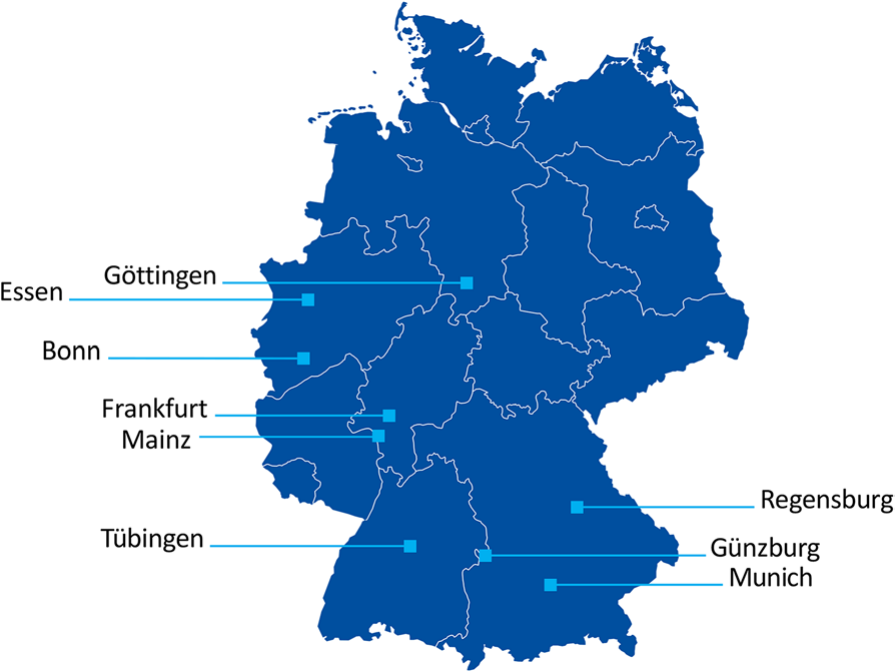
Participating sites in Germany

The trial will be conducted in 9 centers in Germany which must meet the structural and personnel requirements for performing the planned regular trial-related investigations (Fig. 2).

### Eligibility criteria {10}

#### Inclusion criteria

- Male or female subjects, aged ≥ 18 years and ≤ 80 years
- Aneurysmal subarachnoid hemorrhage
- WFNS grades 4 or 5 at admission
- DC can be performed within the first 24 h after symptoms of hemorrhage have started

#### Exclusion criteria

- Moribund patient (mRS ≥ 4 before subarachnoid hemorrhage) due to other illnesses
- SAH due to any other cause than aneurysm rupture (e.g., traumatic, arteriovenous malformation (AVM), fistula, dissection)
- Patients with foreseeable difficulties to perform the follow-up adequately
- Any condition that, in the judgment of the investigator, could impose hazards to the patient if study therapy is initiated or affect the participation of the patient in the study
- Patients with obvious evidence of irreparable brainstem or thalamic injury

### Who will take informed consent? {26a}

As determined by the inclusion criteria, patients that are eligible for the trial will be in a reduced state of consciousness and therefore unable to give informed consent. To protect the vulnerable patient population treated within the trial, we will perform the inclusion of the patients as regularly done for patients unable to give consent. This will include a second opinion of an independent physician before inclusion of a patient as well as informed consent by the legal representative of the trial participants and the informed consent by the patient himself in case of an improved mental state.

### Additional consent provisions for collection and use of participant data and biological specimens {26b}

Information on the collection and use of participant data will be integrated into the informed consent sheet.

## Interventions

### Explanation for the choice of comparators {6b}

Besides the aneurysm treatment in order to prevent re-bleeding and the administration of oral nimodipine as well as intensive care unit (ICU) observation and therapy, there is no causal/outcome-improving therapy available by now. The control group receives therefore the best medical treatment following actual guidelines as stated above. The control group will not be harmed in order to the randomization. Patients in the control group may undergo a secondary DC for therapy-refractory increased intracranial pressure. The experimental group will receive the best medical treatment as well as the pDC.

### Intervention description {11a}

#### Experimental group

- All patients in the study arm will undergo a pDC of at least 12 cm combined with the best possible neuro-intensive medical therapy according to the international guidelines. pDC has to be performed within 24h after ictus.

#### Control group

- Patients will receive the best possible neuro-intensive medical therapy according to the international guidelines. It may include a secondary DC as ultima ratio therapy for reducing increased intracranial pressure.

### Criteria for discontinuing or modifying allocated interventions {11b}

Subjects may withdraw from the trial at any time at their own request without stating the reason(s) for withdrawal. They will experience no disadvantage as a result of this decision and no alternative therapy will be withheld by the investigator.

Moreover, subjects may also be withdrawn at any time at the discretion of the investigator for safety, behavioral, or administrative reasons, e.g.:

- Medically indicated
- Continuation is unacceptable because the risks outweigh the benefits
- Lack of compliance of the subject
- Significant protocol violations
- Logistical reasons (e.g., subject changes his/her doctor or hospital or moves to another location)

### Strategies to improve adherence to interventions {11c}

Not applicable, as interventions are performed early after poor-grade SAH while patients are unconscious.

### Relevant concomitant care permitted or prohibited during the trial {11d}

There are no specific treatments that are prohibited during the trial. All patients receive standard SAH therapy.

### Provisions for post-trial care {30}

Every subject participating in the trial is insured against any trial-related illness/injuries pursuant to the legal requirements, which may occur during the trial.

The investigator will inform the subject of the existence of the insurance, including the obligations arising from it.

### Outcomes {12}

#### Primary outcome

Comparison of the combination of mortality and severe disability between study arms, assessed by mRS between both study arms 12 months after the subarachnoid hemorrhage, dichotomized in “favourable” (mRS 0–4) and “unfavourable” (mRS 5–6) outcome.

#### Secondary outcome

a) Analysis of mortality at 7 and 90 days and at 12 months
  b) Comparison of date of death in patients with pDC vs. no pDC after SAH
  a) Comparison of length of ICU -stay in patients with pDC vs. no pDC after SAH
  b) Comparison of length of hospital stay in patients with pDC vs. no pDC after SAH
Evaluation of the rate of secondary DC due to intractably raised ICP
Evaluation of the use of duraplasty after craniectomy
Evaluation of the correlation between size of craniectomy and clinical outcome
Rate of complications due to craniectomy assessed by AEs/SAEs
Analysis of mRS in patients with pDC vs. no pDC before SAH and 10–14 days, 30 days, 90 days, 180 days, and 2 years (long-term follow-up) after SAH
Analysis of SF36 scores and EQ-5D scores in patients with pDC vs. no pDC 12 months after SAH

### Participant timeline {13}

**Table:** 

	Visit 1 Screening	Visit 2	Visit 3	Visit 4	Visit 5	Visit 6	Visit 7	Visit 8	Visit 9 End of study (EOS)	Visit 10 Long-term FU
Visit window		Max. 24h after SAH	24h after the aneurysm repair	10–14d	End of ICU therapy	30d ± 7d	90d ± 14d	180d ± 14d	12mo ± 30d	24mo ± 30d
Informed consent	√									
Including/excluding criteria	√									
Demographic data	√									
Medical history/anamnesis	√									
CCT or MRI	√		√	√			√			
Vital signs	√		√	√						
Randomization	√									
Decompressive craniectomy		√								
Best medical treatment	√	√	√	√	√	√				
Details of ICU therapy^3^/length of hospital stay					√				√	
Replacement of bone flap							√			
Physical examination^1^	√			√		√	√		√	
mRS assessment in the clinic	√^4^			√		√^2^	√^2^			
Telephone interview incl. mRS^2^						√	√	√	√	√
Documentation of routine laboratory^5^	√			√						
QoL questionnaires (SF36 and EQ-5D)									√	
AEs/SAEs		√	√	√		√	√	√		

^1^Physical examination in visits 7 and 9 only if reasonable for the patient

^2^mRS score to be assessed at the clinic if the patient and next of kin attend visit 6 and visit 7; otherwise, the score is assessed by a telephone interview

^3^Details of ICU therapy: days of ICU therapy/days of sedation/days of hypertension therapy for vasospasm therapy/days of increased ICP (ICP > 15)

^4^mRS score before SAH

^5^Gamma-GT, hemoglobin, Quick, creatinine, leukocytes, c-reactive protein (according to local clinical routine)

### Sample size {14}

The sample size/power calculation is based on a retrospective single-center study and literature review [14].

In this single-center study, 38 patients received pDC and 49 received the best medical treatment, whereas 14 of them underwent secondary DC due to intractably elevated ICP. After 2 years, 10 out of 38 (26%) patients who underwent pDC achieved a favourable outcome compared with 10 out of 49 (20%) patients in the control group (*p*=0.61). In a systematic review of the literature and after selection, 64 out of 131 patients who underwent pDC (49%) achieved a favourable functional long-term outcome.

Simulation analyses showed that the planned Mantel-Hanzel test will have a power of 80% to detect an improvement of the rate of patients with the favorable outcome from 30% under the control intervention to 50% under pDC if the sample size is 102 patients per group. The simulations were performed, assuming minimum six centers. To account for the loss of information due to possible dropouts, 216 patients will be included in the study.

### Recruitment {15}

As SAH is an emergency, no designed recruitment strategy could be implemented. Thus, every patient suffering an aneurysmal SAH and being admitted to one of the participating hospitals (see above) will be screened for eligibility.

## Assignment of interventions: allocation

### Sequence generation {16a}

The allocation to the treatment group will be performed electronically. The subject will be enrolled into the study using the randomization tool of the electronic case report form (eCRF) system called RedCap. The randomization is performed blockwise in a 1:1 ratio to experimental intervention versus control intervention. The randomization will be stratified by trial site.

### Concealment mechanism {16b}

The allocation to the treatment group is concealed by the usage of an electronic randomization tool in the eCRF system.

### Implementation {16c}

The allocation sequence is generated randomly by the computer-based “RedCap” randomization tool implemented by the statistician of the Clinical Study Core Unit Bonn (SZB). After input of all data necessary for randomization (inclusion/exclusion criteria, demographic data), the electronic system displays the randomization result.

## Assignment of interventions: blinding

### Who will be blinded {17a}

Randomization will be open, and only raters for assessment of mRS will be blinded.

### Procedure for unblinding if needed {17b}

Not applicable.

## Data collection and management

### Plans for assessment and collection of outcomes {18a}

Assessment of the initial mRS as well as the baseline characteristics, outcome, and other trial data will be performed by the local investigator supported by a study nurse (e.g., laboratory parameters etc.) and will be documented in the eCRF.

### Plans to promote participant retention and complete follow-up {18b}

Contact data (e.g., address, phone numbers) for each participant and their next of kin (if allowed) will be obtained after recruitment to ensure complete follow-up. We will reschedule phone calls if the participant requests this. Most of the other data (e.g., vital parameters, length of ICU stay, etc.) will be provided by the hospital information system collected in accordance with the indispensable (ICU) treatment.

### Data management {19}

Data management of the study will be carried out by the SZB (section IMBIE (see below)). The study data is recorded and stored in a suitable, validated Clinical Data Management System (CDMS). Details on data management (procedures, responsibilities, data corrections, if any, which may be made by Data Management staff themselves, etc.) will be described in a data management plan prior to the trial. During the trial, the performance of data management and any deviations from the data management plan will be documented in a data management report. Before any data entry is performed, the trial database will be validated and the technical specifications of the database will be documented in a variable plan.

### Confidentiality {27}

The collection, transmission, archiving, and evaluation of personal data in this clinical trial are performed according to local applicable laws (Data Protection Act, General Data Protection Regulation). Prior to trial participation, each subject must be informed by the investigator about the purpose and extent of the collection and use of personal data, particularly medical data, and must give written informed consent.

The subjects must be informed that any subject-related data in this trial are handled confidentially and will be captured in pseudonymized form (subject ID number for the trial, year of birth) and will only be transmitted to the project leaders/data monitoring safety board for scientific and adverse event evaluation as well as the responsible ECs of the trial sites for verifying the proper conduct of the trial and for assessment of trial results and adverse events. During monitoring, audits, or inspections, representatives of the sponsor (monitor, auditor) or of the local regulatory authority(ies) must have direct access to personal data. In this case, the investigator is released from confidential medical communication.

### Plans for collection, laboratory evaluation, and storage of biological specimens for genetic or molecular analysis in this trial/future use {33}

Not applicable as no biological specimens will be collected or used for genetic or molecular analysis in this trial.

## Statistical methods

### Statistical methods for primary and secondary outcomes {20a}

Statistical analysis will be performed at the Institute of Medical Biometry, Informatics and Epidemiology (IMBIE) at the University of Bonn Medical Center. The rate of a favorable outcome will be compared between the treatment groups with a two-sided Mantel-Hanzel test at a level of 5%, stratifying for centers. The overall effect of therapy will be assessed by the calculation of a Mantel-Hanzel overall odds ratio with 95% confidence limits. For this analysis, the outcome of all patients with missing data for the primary outcome at 12 months will be counted as unfavorable.

This analysis will be repeated based on the PP population. Relevant differences between the results of ITT and PP analysis have to be critically discussed.

Mortality will be analyzed by calculating Kaplan-Meier estimators for survival in both treatment groups. Mortality after 7 and 90 days and after 12 months will be derived from the Kaplan-Meier estimators with 95% confidence limits.

Additional details of the statistical analysis will be specified in the statistical analysis plan of the trial.

### Interim analyses {21b}

Not applicable. No interim analysis is planned.

### Methods for additional analyses (e.g. subgroup analyses) {20b}

Not applicable. No additional analyses are planned.

### Methods in analysis to handle protocol non-adherence and any statistical methods to handle missing data {20c}

In case of missing values, multiple imputation methods will be applied for the mRS score and the imputed values will be used to determine the dichotomized outcome. In addition, a sensitivity analysis will be performed treating the patients with missing score values as having an “unfavourable” outcome and also treating only the complete cases.

For analysis of the overall effect of therapy, the outcome of all patients with missing data for the primary outcome at 12 months will be counted as unfavorable.

### Plans to give access to the full protocol, participant-level data, and statistical code {31c}

Individual participant data will be available on reasonable request. Individual participant data (including data dictionaries) that underlie results concerning primary or secondary endpoints reported in a published scientific article will be shared on demand after deidentification. The data will be shared beginning 6 months and ending 3 years following article publication. Data are made available to researchers after a methodologically sound scientific proposal has been submitted to the coordinating investigator and a steering committee, consisting of the coordinating investigator, the representative of the coordinating investigator, and a SZB member, has approved the proposal, and a data access agreement has been signed. The study protocol and the informed consent forms will be made available on demand.

## Oversight and monitoring

### Composition of the coordinating center and trial steering committee {5d}

To ensure accurate, complete, consistent, and reliable data, the investigator’s site(s) and trial procedures will be monitored by a representative of the project leader. The project leader’s representative will visit the site:

- To evaluate the progress and recruitment of the trial
- To review the source documents and CRFs for protocol compliance, accuracy, and validation
- To assess facilities and equipment
- To check for protocol compliance
- To assure the AE documentation

Source data verification will be performed in order to verify the accuracy and completeness of the entries on the case report form (CRF) by comparing them with the source data and to ensure and increase the quality of the data. All data which are subject to SDV must have been entered in the medical record or, in the case of source documents, enclosed with the medical record. The investigators will afford the CRA access to the medical records for the performance of SDV.

The frequency and scope of the monitoring visits will be defined in the Monitoring Plan for this trial which also includes the extent of source data verification that is required.

### Composition of the data monitoring committee, its role, and reporting structure {21a}

The Data Safety Monitoring Board (DSMB) is an independent committee monitoring the study progress of the safety of trial participants and the quality of the collected data (by monitoring reports) and should make recommendations on the discontinuation, modification, or continuation of the trial. The tasks of the DSMB in the trial are the following:

The DMSB will review all SAEs of all patients so far recruited to the trial regularly.

The coordinating investigator can ask the DSMB at any time to review any data from the trial and to decide whether to proceed with the trial without changes, to modify the trial, or to stop the trial entirely. The data provided to the DSMB for review may also include so far unmonitored data.

The DMSB is free to suggest any modifications regarding the trial (e.g., stopping of the trial, modifications of the protocol).

The DSMB will include at least 3 members with experience in the conduct of the study and individual expertise in the field of neurology and biometrics. Further details concerning members, function, reports, and modalities of meetings are provided by the DSMB Charter of this trial.

The Data Safety Monitoring Board (DSMB) has been appointed to review the conduct and results of this trial at 2 enrolment landmarks (after 50 and 100 of the patients have been enrolled). The DSMB is charged with reviewing safety data in both arms of the trial. The DSMB will be empowered to stop the study for evidence of harm but not for evidence of lack of efficacy. The DSMB is also asked to offer perspective on any therapeutic or diagnostic testing advances that may occur during the course of the trial that may influence the outcome. If protocol modifications are warranted, close consultation among the DSMB, the SZB staff, and the study leadership will be required. Further details are provided in a separate DSMB charter.

### Adverse event reporting and harms {22}

Any AE defined in the trial protocol as relevant for the evaluation and analysis of the clinical trial has to be documented in the CRF on the respective adverse event report form. The following criteria will be assessed: type, beginning, end, and outcome of the event (recovered, improved, unchanged, recovered with sequelae, worsened death, unknown). Pregnancy is not an exclusion criterion in this lifesaving treatment study.

All safety evaluations will be performed on the ITT population, which consists of all patients who were randomized. Frequency tables and/or summary statistics will be provided as described below for the observed adverse events (AEs) and for patients having a respective AE:

- Per system organ classes
- Per preferred terms
- Therapy-related AEs
- Serious AEs related to therapy

### Frequency and plans for auditing trial conduct {23}

In accordance with ICH GCP, this trial may be selected for audit by representatives of the project leader.

The investigator agrees to give the auditor access to all relevant documents for review and to support the project leader to solve possible audit findings concerning the trial conduct at the respective site.

After every audit, the auditee(s) will receive an audit confirmation by the auditor. This document has to be filed together with the trial documentation.

At the end of the trial, a copy of the audit certificate(s) will be included in the final report.

### Plans for communicating important protocol amendments to relevant parties (e.g., trial participants, ethical committees) {25}

The project leaders can make general amendments to the protocol after the clinical trial has started. These may be of an administrative nature (logistical/administrative amendments) or substantial.

Substantial amendments are changes that likely affect and/or change:

- The safety of the persons concerned
- The interpretation of the scientific trial documents or the scientific informational value of the trial results
- The nature of management or conduct of the clinical trial (e.g., change of project leaders), require a new favorable opinion by the Ethics Committee

The clinical trial may only be continued when a favorable opinion has been obtained from the competent ethics committee.

If applicable, an updated informed consent form has to be signed by all subjects enrolled in the trial who are affected by the amendment.

Amendments which only have to be approved by the EC (e.g., changes in an advertisement for subjects to participate in the trial or changes in facilities for the trial) also will be notified to the CA with the comment “For information only.” Similarly, the EC will be informed of any substantial amendments for which only the CA is responsible (e.g., quality data).

If administrative protocol changes (e.g., change of monitoring, telephone numbers) are necessary, the EC and CA will be notified only.

## Dissemination plans {31a}

The results will be reported in peer-reviewed scientific journals and presented in meetings/conferences. The results will also be shared with participants, healthcare professionals, and the public through lectures or science handbooks if realizable or on demand.

The right of publication rests primarily with the sponsor, the coordinating investigator, and the other investigators involved. All data collected in connection with the clinical trial will be treated in confidence by the sponsor/coordinating investigator and all others involved in the trial, until publication. Interim data and final results may only be published (orally or in writing) with the agreement of the sponsor, the coordinating investigator, and the other investigators. This is indispensable for a full exchange of information between the above-named parties, which will ensure that the opinions of all parties involved have been heard before publication. The agreement, which does not include any veto right or right of censorship for any of the parties involved, may not be refused without good reason.

Specific regulations concerning the publication policy in the applicable contracts will precede this trial protocol in any case.

## Discussion

SAH is a devastating disease with high morbidity and mortality, especially in poor-grade SAH. DC is widely used in neurosurgical conditions causing intractably elevated ICP as a lifesaving procedure with reasonable side effects. While DC is performed in patients suffering from SAH at variable time points with diverse underlying reasons for intractably elevated ICP (e.g., brain swelling, re-bleeding, infarction), the effect of an early DC (i.e., pDC) in poor-grade SAH has only been investigated in retrospective studies so far. While the results are encouraging, their retrospective and uncontrolled nature does not allow any conclusion regarding the effectiveness on reducing mortality and severe disability in poor-grade SAH. The PICASSO trial will be the first prospective trial to analyze the effect of pDC on outcomes in poor-grade SAH.

## Trial status

Recruitment has begun in October 2019 and will be completed by September 2023.

## Supplementary Information

**Additional file 1.** Model consent form given to participants and authorized surrogates.

## Data Availability

The database of the trial is completely in the hand of the SZB and therefore separated from the investigational and coordinating sites. After database closure, the responsible biostatistician will provide the calculations (details of the analyses will be determined in the statistical analysis plan) without interaction with the coordinating investigator.

## Abbreviations

AE: Adverse event
SAH: Aneurysmal subarachnoid hemorrhage
AVM: Arteriovenous malformation
CCT: Cranial computer tomography
CRA: Clinical research associate
DC: Decompressive craniectomy
DSMB: Data Safety Monitoring Board
eCRF: Electronic case report form
EC: Ethics Committee
EOS: End of study
EQ-5D: European Quality of Life-5 Dimensions
FU: Follow-up
GCP: Good Clinical Practice
ICH: International Conference on Harmonization
ICP: Intracranial pressure
ICU: Intensive care unit
IIT: Investigator-initiated trial
IMBIE: Institute of Medical Biometry, Informatics and Epidemiology
ITT: Intention-to-treat
MRI: Magnetic resonance imaging
mRS: Modified Rankin Scale
pDC: Primary decompressive craniectomy
PP: Per-protocol (population)
QoL: Quality of life
SAE: Serious adverse event
SDV: Source data verification
SF36: Short Form (36) Health Survey
SZB: Study Center Bonn
TBI: Traumatic brain injury
WFNS: World Federation of Neurosurgical Societies
