# Supplementary material for: PrImary decompressive Craniectomy in AneurySmal Subarachnoid hemOrrhage (PICASSO) trial: study protocol for a randomized controlled trial
Source: Trials. 2022 Dec 20;23:1027. doi: 10.1186/s13063-022-06969-4 (PMC9764529; doi:10.1186/s13063-022-06969-4)
Supplement: Supplementary file 1 — Additional file 1. Model consent form given to participants and authorized surrogates. [file 13063_2022_6969_MOESM1_ESM.zip › renamed_562edR1.pdf]

Nachträgliche Patienteninformation und –einwilligung zur Durchführung einer klinischen Studie mit volljährigen einwilligungsfähigen Patienten[1]

PICASSO

**Studientitel:**	**Primäre dekompressive Kraniektomie bei aneurysmatischer Subarachnoidalblutung**
**Studienkurztitel:**	**PICASSO**
**Prüfplan Code:**	**NCH_201702_PICASSO**
**DRKS-ID.:**	**DRKS00017650**

Studienzentrum und Studienarzt:

Prof. Erdem Güresir
Universitätsklinikum Bonn
Klinik und Poliklinik für Neurochirurgie
Gebäude 81
Venusberg-Campus 1
53127 Bonn

Sehr geehrte Patientin, sehr geehrter Patient,

während Sie nicht einwilligungsfähig waren, wurden Sie in die oben genannte Studie eingeschlossen. Es wurde angenommen, dass Sie eingewilligt hätten, wenn Sie dazu in der Lage gewesen wären (mutmaßlicher Wille).

Wir möchten Sie bitten, nun zu entscheiden, ob Sie in die Teilnahme an der Studie einwilligen oder diese ablehnen. In dieser Information finden Sie alles Wesentlich zu der Studie.

Bitte lesen Sie diese Information sorgfältig durch. Ihre Ärztin/Ihr Arzt wird mit Ihnen über die Studie sprechen und Ihre Fragen beantworten.

Es werden 216 Patienten an 9 Studienzentren eingeschlossen.

Bei uns sollen ca. 20 Personen an der Studie teilnehmen.

Die Studie wurde von Uniklinikum Bonn geplant und wird in Kooperation mit Universitätsklinikum Tübingen, Universitätsklinikum Regensburg, Universitätsklinikum Frankfurt am Main, Universitätsklinikum Essen, Universitätsmedizin Göttingen, Klinik für Neurochirurgie BKH Günzburg, Universitätsklinikum Mainz, Universitätsklinikum Bonn, TU München durchgeführt.

Die Studie wird durch das “Förderinstrument Klinische Studien der Kommission für Klinische Studien“ der Medizinischen Fakultät der Universität Bonn gefördert.

[1]Im Rahmen dieses Textes schließt die männliche Bezeichnung stets die weibliche Bezeichnung mit ein.

Eine unabhängige Ethikkommission hat die Studie geprüft und im Rahmen der Beratung keine Einwände gegen die Durchführung erhoben.

Die Teilnahme an der Studie ist freiwillig. Wenn Sie nicht teilnehmen wollen oder wenn Sie später Ihre Einwilligung widerrufen, werden Ihnen daraus keine Nachteile entstehen.

Was wir mit dieser Studie erreichen wollen:

Hirnblutungen treten auf, wenn Blutgefäße im Schädelinneren verletzt werden. Dies kann vorkommen, wenn Blutgefäße im Gehirn geschädigt sind. Hier kommen unter anderem krankhafte Erweiterung der Wand einer Schlagader (Aneurysma) ursächlich in Frage. Wenn eine solche Erweiterung (Hirnaneurysma) reißt, läuft Blut in den mit Flüssigkeit gefüllten Raum, der das Gehirn umgibt. Der Fachbegriff für diesen Bereich lautet Subarachnoidalraum. Wenn es dort hineinblutet, spricht man deshalb von einer Subarachnoidalblutung.

Wir untersuchen die Möglichkeit einer frühzeitigen chirurgischen Therapie einer der Hauptkomplikationen bei der schweren Form der aneurysmatischen Hirnblutung. Sie leiden an dieser Hirnblutung. Ihr Arzt wird Sie informieren, welche Behandlungen bereits durchgeführt wurden und welche weiteren Behandlungsmöglichkeiten bestehen.

Die Form der Hirnblutung, um welche es bei der PICASSO-Studie geht (aneurysmatische Subarachnoidalblutung) macht 2-5 % aller Schlaganfälle aus. Nur etwa 20 % der Patienten mit einer oben beschriebenen Hirnblutung überleben und erreichen anschließend nach 3-6 Monaten ihre vorherige Lebensqualität.

Durch die Hirnblutung kann es zu schwerwiegenden Ausfällen des Nervensystems kommen. Es können auch dauerhaften Behinderungen entstehen. Die Hirnblutung Schwellungen des Gehirns verursachen und das Gehirn kann dauerhaft geschädigt werden. Um diesen Druck zu entlasten kann die Entfernung eines Teils des Knochendeckels (Entlastungskraniektomie) dazu dienen, den erhöhten Hirndruck effektiv zu senken. Der Druck auf das angrenzende Hirngewebe wird dabei reduziert.

Die Entlastungskraniektomie, also das Entfernen des Schädelknochens stellt in der Neurochirurgie ein bewährtes Standardverfahren dar. Es wird schon eingesetzt bei Schlaganfall, der durch eine verminderte Durchblutung des Gehirns ausgelöst wurde. Auch bei schwerstem Schädel-Hirn-Trauma wird es eingesetzt, um ebenfalls effektiv einen erhöhten Hirndruck zu senken. Menschen, die eine Hirnblutungen erleiden sterben oft. Durch die vorrangige Entfernung eines Teils des Knochendeckels bei Patienten mit einer Hirnblutung, soll mit dieser Studie untersucht werden, ob die Sterblichkeit reduziert werden kann. Außerdem soll untersucht werden, ob das Behandlungsergebnis langfristig verbessert werden kann.

Es gibt bereits Studien, wo die Auswirkung der Entfernung des Schädelknochens (innerhalb von 24 Stunden nach Auftreten der Blutung) untersucht wurde im Hinblick auf die Sterblichkeitsrate und den Behinderungsgrad. Vor allem bei schwerst betroffenen Patienten mit der beschriebenen Hirnblutung. Diese Studien sind jedoch im Nachhinein mit bereits vorhandenem Datenmaterial erhoben worden. Mit der PICASSO-Studie soll vorrausschauend überprüft werden, ob eine Behandlungsmethode Vorteile im Hinblick auf die Sterblichkeitsrate und den Behinderungsgrad darstellt, oder ob beide gleichwertig einzuschätzen sind.

Der Schädelknochen soll frühzeitig entfernt werden. Damit wird der Druck im Schädelinneren entlastet und die mögliche Schädigung kann dabei vermindert werden. Die Entfernung des Schädelknochens kann

so eventuell die Sterblichkeit und die Rate der Langzeitbehinderung verkleinern. Mit dieser klinischen Studie wollen wir die Wirksamkeit bei diesem Behandlungsvorgehen vergleichen mit den gängigen Maßnahmen. Zu den gängigen Maßnahmen gehört entweder eine erst verzögerte Entlastungskraniektomie (später als 24h nach dem Blutungsereignis), oder keine Entlastungskraniektomie (Standardtherapie).

Beim Entfernen des Schädelknochens wird die äußere Schicht, die das Gehirn umschließt, sichtbar. Das ist die sogenannte harte Hirnhaut. Es gibt vereinzelt Kliniken, die diese harte Hirnhaut verschließen mit einer sogenannten Duraplastik. Sollte das bei der Klinik, die Sie betreut der Fall sein, werden Sie entsprechend darüber informiert. Eine Anwendung der Duraplastik ist klinische Routine. Ihre Klinik wird Sie darüber aufklären.

Wie ist der Ablauf der Studie?

Die Patienten werden nach dem Zufallsprinzip in zwei Gruppen (50% in jeder Gruppe) eingeteilt. Beide Gruppen erhalten eine Therapie, die standardgemäß durchgeführt wird. Eine Aussage, welches die bessere Behandlungsmethode ist, kann bisher nicht beurteilt werden:

Gruppe A: chirurgische Behandlungsgruppe:
Bei Patienten, welche in Gruppe A eingeteilt werden, wird der Schädelknochen innerhalb von 24 Stunden nach der Blutung entfernt. Kombiniert wird die Operation mit der bestmöglichen neurointensiven medizinischen Therapie nach internationalen Richtlinien.

Gruppe B: Kontrollgruppe:
Patienten, welche in Gruppe B eingeteilt werden, erhalten die bestmögliche neurointensive medizinische Therapie nach internationalen Richtlinien. Sollte der Hirndruck anhaltend erhöht sein, kann der behandelnde Arzt entscheiden eine sekundäre Entlastungskraniektomie durchzuführen, also ebenfalls den Schädelknochen zu entfernen.

Die Studie wird für jeden Teilnehmer voraussichtlich 24 Monate dauern.

Studienablauf:

Bitte beachten Sie, dass diese Maßnahmen bei Ihnen zum Teil schon erfolgt sind oder noch weitere davon erfolgen werden. Ihr zuständiger Studienarzt wird Sie darüber informieren.

- Das Leben von Patienten mit Hirnblutungen ist gefährdet. Durch die Hirnblutung kann es zur Störungen der Hirnfunktion kommen. Deshalb werden diese Patienten intensiv überwacht und auch einer Überwachungs- oder Intensivstation behandelt.
- Patienten mit dieser Hirnblutung (aneurysmatischer Subarachnoidalblutung), welche an der klinischen Studie teilnehmen, werden nach dem Zufallsprinzip zugeteilt. Sie wurden entweder in die chirurgische Behandlungsgruppe eingeteilt und der Schädelknochen wurde entfernt, oder es wurde die Standardbehandlung angewendet.
- Es wurde routinemäßig Blut abgenommen und ein Bild vom Kopf gemacht (Computertomographie oder Magnetresonanztomographie). Ihr Gesundheitszustand vor der Hirnblutung wurde anhand eines standardisierten Fragebogens beurteilt.
- Das Entfernen des Schädelknochens muss spätestens 24 Stunden nach Beginn der Blutung erfolgen. Dies trifft bei den Patienten zu, die für diese Behandlunsgruppe ausgewählt wurden. Bei der sogenannten Kraniektomie wird der Schädelknochen entfernt, die harte Hirnhaut eröffnet und so der Druck der Hirnblutung auf das angrenzende Hirngewebe reduziert
- Ca. 24 Stunden nach Versorgung der Blutung wird ein Bild des Kopfes erstellt, ein weiteres Bild wird angefertigt 10-14 Tage nach Blutungsereignis. Dies ist eine routinemäßige Maßnahme und würde auch ohne Teilnahme an der Studie gemacht werden.

- Vor der Entlassung aus der Klinik wird standardmäßig bei diesen Patienten eine klinische Untersuchung durchgeführt.
- 30 Tage nach erlittener Hirnblutung werden die Patienten im Rahmen der Studie telefonisch kontaktiert um einige Fragen zu beantworten (falls die Patienten nicht mehr in der neurochirurgischen Klinik sein sollten). Dieses Telefonat dauert ungefähr 10 min und dient dazu, zu überprüfen wie stark die Patienten im täglichen Leben durch die Hirnblutung beinträchtig werden. Dieses Telefonat wird nach weiteren 3 Monaten (+/- 14 Tagen) nochmals wiederholt. Dabei werden noch zusätzlich Fragen zur Lebensqualität gestellt. Das zweite Telefonat dauert ungefähr 20 Minuten. Nach 12 und 24 Monaten (+/- 30 Tagen) findet nochmals das 20-minütige Telefonat statt. Bei dem Telefonat nach 12 Monaten werden ebenso Fragen zur Lebensqualität erfasst. Das Telefonat kann insgesamt etwa 30 Minuten dauern. Kann der Patient telefonisch nicht selber Auskunft geben, werden Angehörige oder Betreuer befragt.

  Sollten Sie sich in dem Zeitraum, der für die Befragung angedacht ist in der Klinik befinden, so werden die Daten vor Ort erhoben und auf das Telefonat wird verzichtet.
- Drei Monate (+/- 14 Tage) nach der Hirnblutung erfolgt bei den Studienteilnehmern ein Bild des Kopfes (Computertomographie oder Magnetresonanztomographie) von ca. 10 bis 30 Minuten Dauer. Es findet auch eine körperliche Untersuchung mit einer Dauer von ca. 30 Minuten statt. Beide Untersuchungen erfolgen nach Möglichkeit am gleichen Tag. Diese Untersuchungen würden auch unabhängig von der Teilnahme an der Studie durchgeführt.
- Bei Patienten, welchen der Schädelknochen entfernt wurde, erfolgt nach der Abschwellung des Hirns die Wiedereinsetzung des Schädelknochens. Meist kann diese Operation ca. 3 Monate nach der Hirnblutung erfolgen. Für die Operation müssen die betroffenen Studienteilnehmer für ca. 5 Tage stationär in der Klinik aufgenommen werden.
- Bis auf die Telefonate werden nur Untersuchungen durchgeführt, die auch unabhängig von der Teilnahme an der klinischen Studie durchgeführt werden.

Gibt es einen persönlichen Nutzen durch die Teilnahme an der Studie?

Von der Teilnahme an der Studie werden Sie keinen persönlichen Nutzen haben. Die Ergebnisse der Studie können jedoch in Zukunft anderen Menschen helfen.

Welche Risiken sind mit einer Teilnahme an der Studie verbunden?

Die Risiken der Entlastungskraniektomie sind:

- Wundheilungsstörungen (3.5%)
- Infektionen im Bereich der Operationswunde (3.2%)
- Blutungen im Hirn oder im Bereich der Hirnhäute und des Knochens während oder nach der Operation (2-3%)
- Austritt von Gehirnwasser (Nervenwasser-Fistel, 0.6%)

Die Risiken des Wiedereinsetzens des Schädelknochens sind:

- Wundheilungsstörungen (6.1%)
- Infektionen im Bereich der Operationswunde (3.6%)
- Blutungen im Hirn oder im Bereich der Hirnhäute und des Knochens während oder nach der Operation (4.1%)
- Auflösung des wieder eingesetzten Knochens mit möglicher erneuter Operation (4%)
- Austritt von Gehirnwasser (Nervenwasser-Fistel, 1%)
- Ansammlung von Gehirnwasser im Bereich der Hirnhäute (subdurales Hygrom, 1.5%)
- Verschiebung des wieder eingesetzten Schädelknochenlappens (0.5%)

Entstehen zusätzliche Kosten?

Durch die Teilnahme an der Studie entstehen weder Ihnen noch der Krankenkasse zusätzliche Kosten.

Welche Rechte Sie haben, wenn Sie an der Studie teilnehmen:

Ihre Teilnahme an dieser Studie ist freiwillig. Sie können jederzeit die Teilnahme beenden. Sie müssen dies nicht begründen. Es entstehen für Sie dadurch auch keine Nachteile für Ihre medizinische Behandlung oder Ihr Verhältnis zu Ihrem behandelnden Arzt.

Wenn Sie die Einwilligung widerrufen möchten, wenden Sie sich bitte an die Studienleitung oder das behandelnde Personal. Bei einem Widerruf können Sie entscheiden, ob die studienbedingt erhobenen Daten gelöscht werden sollen, oder ob erstellte Aufnahmen vernichtet werden sollen oder weiterhin für die Zwecke der Studie verwendet werden dürfen. Auch wenn Sie einer weiteren Verwendung zunächst zustimmen, können Sie nachträglich Ihre Meinung noch ändern und die Löschung der Aufnahmen verlangen; wenden Sie sich dafür bitte ebenfalls an die Studienleitung oder das Sie behandelnde Personal. Falls Sie die Einwilligung widerrufen, werden Sie zu Ihrer Sicherheit im Rahmen der Studie abschließend medizinisch untersucht.

Ein Ausschluss aus der Studie ist möglich, wenn dies medizinische oder organisatorische Gründe notwendig machen.

Welche Pflichten sind mit der Teilnahme an der Studie für Sie verbunden:

Wenn Sie bei der klinischen Studie weiterhin mitmachen möchten, müssen Sie bestimmte Regeln beachten. Dies ist notwendig für Ihre Sicherheit und Gesundheit. Wir werden Sie dabei so gut wir können unterstützen. Soweit es Ihnen möglich ist, verpflichten Sie sich als Studienteilnehmender:

- den medizinischen Anweisungen seines Studienarztes zu folgen und sich an den Studienplan zu halten.
- Ihren Studienarzt über den Verlauf der Erkrankung zu informieren und neue Symptome, neue Beschwerden und Änderungen im Befinden zu melden.
- Ihren Studienarzt über die gleichzeitige Behandlung und Therapien bei einem anderen Arzt und über die Einnahme von Medikamenten zu informieren. Dabei sind alle Medikamente zu nennen, auch solche, die selbst gekauft sind, für die kein Rezept benötigt wird, oder auch Kräutertees, pflanzliche Arzneien etc. Sie müssen auch Medikamente der Alternativmedizin nennen: Homöopathie, etc.
- Wenn Sie die Pflichten nicht beachten, können Sie die Haftungsansprüche verlieren.

Probandenversicherung und Verfahren im Schadensfall:

Die Teilnahme an der Studie ist versichert. In dem seltenen Fall, dass aufgrund Ihrer Teilnahme an den Untersuchungen zu der Studie Gesundheitsschädigungen aufgetreten sind, werden diese von einer Versicherung abgedeckt. Der Umfang des Versicherungsschutzes ergibt sich aus den Versicherungsunterlagen, die Sie vom Studienarzt ausgehändigt bekommen. Die Höchstersatzleistung pro Patient beträgt maximal 500.000 Euro.

Studienteilnehmerversicherung:

Name des Unternehmens:	**HDI-Gerling Industrieversicherung AG**
Anschrift:	Riethorst 2, 30659 Hannover
Vertreten durch:	Niederlassung Düsseldorf<br>Am Schönenkamp 45, 40599 Düsseldorf
Versicherungsmakler:	Ecclesia mildenberger HOSPITAL GmbH<br>Ecclesiastraße 1- 4, 32758 Detmold
**Telefon:**	**05231/603-6486**
**Telefax:**	**05231/603-606486**
**Versicherungsnummer:**	**57 010323 03010**

Wenn Sie vermuten, dass durch die Teilnahme an der klinischen Studie Ihre Gesundheit geschädigt oder bestehende Leiden verstärkt wurden, müssen Sie dies unverzüglich dem Versicherer direkt anzeigen, gegebenenfalls mit Unterstützung durch Ihren Studienarzt, um Ihren Versicherungsschutz nicht zu gefährden. Sofern Ihr Studienarzt Sie dabei unterstützt, erhalten Sie eine Kopie der Meldung. Sofern Sie Ihre Anzeige direkt an den Versicherer richten, informieren Sie bitte zusätzlich Ihren Studienarzt.

Bei der Aufklärung der Ursache oder des Umfangs eines Schadens müssen Sie mitwirken und alles unternehmen, um den Schaden abzuwenden und zu mindern.

Sie erhalten ein Exemplar der Versicherungsbestätigung einschließlich der Versicherungsbedingungen.

Wir weisen Sie insbesondere auf Punkt 1.4 (zu den Ausschlüssen), Punkt 3.1 (zum Umfang der Leistungen) und Punkt 4.3 sowie Punkt 4.4. (zu Ihren Obliegenheiten) hin.

Eine zusätzliche Wege-Unfall-Versicherung wurde nicht abgeschlossen.

Was geschieht mit den Daten:

Die Mehrzahl der erhobenen Daten wird im Rahmen Ihrer klinischen Betreuung/Behandlung erhoben bzw. wurde bereits vor der Studie erhoben; diese sind bzw. werden personenbezogen gespeichert.

Nur wenige Daten werden speziell im Rahmen der Studie erhoben; diese werden in pseudonymisierter Form im Institut für Medizinische Biometrie, Informatik und Epidemiologie des Universitätsklinikums Bonn gespeichert und ausgewertet. Pseudonymisiert bedeutet, dass keine Angaben von Namen oder Initialen verwendet werden, sondern nur ein Nummer- und/oder Buchstabencode eventuell mit Angabe des Geburtsjahres.

Zugriff auf diese Daten haben nur Mitarbeiter der Studie. Diese Personen sind zur Verschwiegenheit verpflichtet. Die Daten sind vor fremdem Zugriff geschützt. Sie können darüber entscheiden, ob Ihr Hausarzt oder andere behandelnde Ärzte über Ihre Teilnahme an dieser klinischen Studie informiert werden sollen, um dies bei Ihrer weiteren Behandlung ggf. zu berücksichtigen.

Aufgrund gesetzlicher Regelungen haben autorisierte Dritte ein Recht auf Einsichtnahme in Ihre Daten. Die Einsichtnahme erfolgt nur im Rahmen der gesetzlich geregelten Aufgaben des Einsicht nehmenden, nämlich zum Zweck der Überprüfung der Daten. Diese Personen sind ebenfalls zur Verschwiegenheit verpflichtet.

Die im Rahmen der Studie erhobenen, personenbezogenen Daten werden nach Erreichen des Studienziels gelöscht, soweit gesetzliche Vorgaben nicht längere Fristen vorsehen.

Bei der Veröffentlichung von Studienergebnissen wird aus den Daten nicht hervorgehen, wer an dieser Studie teilgenommen hat. Ein Bezug zu Ihrer Person kann nicht hergestellt werden.

Einzelheiten zur Verarbeitung Ihrer Daten, insbesondere zur Möglichkeit eines Widerrufs, entnehmen Sie bitte der Einwilligungserklärung, die im Anschluss an diese Patienteninformation abgedruckt ist.

Sind mit der Datenverarbeitung Risiken verbunden?

Bei jeder elektronischen Erhebung, Speicherung und Auswertung von Daten bestehen Vertraulichkeitsrisiken (z.B. die Möglichkeit, die betreffende Person zu identifizieren). Diese Risiken lassen sich nicht völlig ausschließen und steigen, je mehr Daten miteinander verknüpft werden können. Der Initiator der Studie versichert Ihnen, alles nach dem Stand der Technik Mögliche zum Schutz Ihrer Privatsphäre zu tun und Daten nur an die Studienzentrale SZB Bonn des Universitätsklinikums Bonn weiterzugeben, die ein geeignetes Datenschutzkonzept vorweisen können. Medizinische Risiken sind mit der Datenverarbeitung nicht verbunden.

Kann ich meine Einwilligung widerrufen?

Sie können Ihre jeweilige Einwilligung jederzeit ohne Angabe von Gründen schriftlich oder mündlich widerrufen, ohne dass Ihnen daraus ein Nachteil entsteht. Wenn Sie Ihre Einwilligung widerrufen, werden keine weiteren Daten mehr erhoben. Die bis zum Widerruf erfolgte Datenverarbeitung bleibt jedoch rechtmäßig.

Sie können im Fall des Widerrufs auch die Löschung Ihrer Daten verlangen. Die Studiendaten, die bereits anonymisiert und somit nicht mehr Ihrer Person zugeordnet werden können, werden im Rahmen der Studie weiterverwendet.

An wen können Sie sich wenden:

Sie haben stets die Gelegenheit zu weiteren Beratungsgesprächen mit dem auf Seite 1 genannten oder einem anderen Studienarzt und dem medizinischen Personal, um Fragen im Zusammenhang mit der klinischen Studie zu klären. Auch Fragen, die Ihre Rechte und Pflichten als Teilnehmer an der klinischen Studie betreffen, werden gerne beantwortet.

Verantwortlich für die gesammelten Studiendaten ist der Projektleiter der Studie.

Prof. Dr. med. Erdem Güresir

Stellvertretender Direktor und leitender Oberarzt der Universitätsklinik für Neurochirurgie

Venusberg-Campus 1, 53127 Bonn

Telefon: +49 228 287 11350

E-Mail: PICASSO@ukbonn.de

UNIVERSITÄT BONN · ukb universitäts klinikumbonn · SZB Studienzentrum Bonn Studienzentrale · Klinik und Poliklinik für Neurochirurgie

Nachträgliche Einwilligungserklärung

**Studientitel:**	**Primäre dekompressive Kraniektomie bei aneurysmatischer Subarachnoidalblutung**
**Studienkurztitel:**	**PICASSO**
**Prüfplan Code:**	**NCH_201702_PICASSO**
**DRKS-ID.:**	**DRKS00017650**

Studienzentrum und Studienarzt:

Prof. Erdem Güresir
Universitätsklinikum Bonn
Klinik und Poliklinik für Neurochirurgie
Gebäude 81
Venusberg-Campus 1
53127 Bonn

Ich ______________________________

Name des Patienten in Druckbuchstaben

geb. am ____________________ Teilnehmer-Nr. ____________________

erkläre, dass ich die Patienteninformation zur o.g. wissenschaftlichen Untersuchung und diese Einwilligungserklärung erhalten habe.

☐ bin im Nachhinein in einem persönlichen Gespräch durch den Studienarzt ausführlich und verständlich über Studienvorgehen und die Vergleichstherapie, Wesen, Bedeutung und Tragweite der klinischen Studie sowie die sich für mich daraus ergebenden Anforderungen aufgeklärt worden. Ich habe darüber hinaus den Text der Patientenaufklärung und dieser Einwilligungserklärung sowie die hier nachfolgend abgedruckte Datenschutzerklärung gelesen und verstanden.

☐ Ich hatte ausreichend Zeit und Gelegenheit, Fragen zu stellen und mich zu entscheiden. Alle aufgetretenen Fragen über die Durchführung der klinischen Studie wurden mir vom Studienarzt zufrieden stellend beantwortet.

☐ Ich weiß, dass ich jederzeit und ohne Angabe von Gründen meine freiwillige Einwilligung zur Teilnahme an der Studie zurückziehen kann (mündlich oder schriftlich), ohne dass mir daraus Nachteile für meine medizinische Behandlung entstehen.

Möglichkeit zur Dokumentation zusätzlicher Fragen seitens des Patienten oder sonstiger Aspekte des Aufklärungsgesprächs:

____

____

____

____

Ich hatte ausreichend Zeit, mich für eine Fortführung der Studie zu entscheiden.

Datenschutz:

Mir ist bekannt, dass bei dieser wissenschaftlichen Studie personenbezogene Daten, insbesondere medizinische Befunde über mich erhoben, gespeichert und ausgewertet werden sollen. Die Verwendung der Angaben über meine Gesundheit erfolgt nach gesetzlichen Bestimmungen und setzt vor der Teilnahme an der wissenschaftlichen Studie folgende freiwillig abgegebene Einwilligungserklärung voraus. Diese informierte Einwilligungserklärung ist die Rechtsgrundlage gemäß Datenschutz-Grundverordnung Artikel 6 Absatz 1a) zur Verarbeitung Ihrer Daten. Das heißt, ohne die nachfolgende Einwilligung kann ich nicht an der wissenschaftlichen Studie teilnehmen.

1. Ich willige ein, dass im Rahmen dieser klinischen Studie personenbezogene Daten über mich, insbesondere Angaben über meine Gesundheit und meine ethnische Herkunft, über mich, wie in der Informationsschrift beschrieben erhoben und in Papierform sowie auf elektronischen Datenträgern im Studienzentrum (S.1) aufgezeichnet werden. Zu diesem Zweck entbinde ich die mich behandelnden Ärzte von der ärztlichen Schweigepflicht.

   Soweit erforderlich, dürfen die erhobenen Daten pseudonymisiert (verschlüsselt) weitergegeben werden:

   a) an die Leitung der klinischen Studie oder von diesem beauftragten Stellen (z.B. Studienzentrale SZB Bonn) zum Zweck der wissenschaftlichen Auswertung,
2. Außerdem erkläre ich mich damit einverstanden, dass autorisierte und zur Verschwiegenheit verpflichtete Beauftragte des Auftraggebers in meine personenbezogenen Daten, insbesondere meine Gesundheitsdaten, Einsicht nehmen, soweit dies für die Überprüfung der ordnungsgemäßen Durchführung der wissenschaftlichen Untersuchung notwendig ist. Für diese Maßnahme entbinde ich den Studienarzt von der ärztlichen Schweigepflicht.
3. Ich erkläre mich damit einverstanden, dass meine in Zusammenhang mit der Studie erhoben Daten nach Beendigung oder Abbruch der Studie höchstens 10 Jahre aufbewahrt werden. Danach werden meine personenbezogenen Daten gelöscht, soweit nicht gesetzliche oder satzungsmäßige Aufbewahrungsfristen entgegenstehen.
4. Die gesetzlichen Bestimmungen enthalten nähere Vorgaben für den erforderlichen Umfang der Einwilligung in die Datenerhebung und -verwendung. Gemäß der europäischen Datenschutzgrundverordnung (EU-DSGVO) haben Sie das Recht auf:
   - Auskunft über die Verarbeitung Ihrer Daten,
   - Berichtigung oder Löschung Ihrer Daten,

- Einschränkung der Verarbeitung (nur noch Speicherung möglich),
- Widerspruch gegen die Verarbeitung,
- Datenübertragbarkeit (die Übermittlung der Daten an Sie oder – soweit technisch möglich – an eine andere von Ihnen benannte Stelle),
- Widerruf Ihrer gegebenen Einwilligung mit Wirkung auf die Zukunft,
- Überlassung einer unentgeltlichen Kopie Ihrer personenbezogenen Daten,
- Beschwerde bei der Datenschutzaufsichtsbehörde.

5. Ich bin darüber aufgeklärt worden, dass ich jederzeit die Teilnahme an der klinischen Studie beenden kann. Mein Widerruf zur Studienteilnahme hat für mich keinerlei negative Auswirkungen.

   Ich bin willige ein **ja ☐/ nein ☐ (bitte ankreuzen)**, dass im Falle meines Widerrufs zur Teilnahme an der klinischen Studie die bis zu diesem Zeitpunkt gespeicherten Daten weiterhin verwendet werden dürfen. Falls keine Einwilligung erfolgt, werden im Fall meines Widerrufs die Daten vollständig gelöscht.

Der Übermittlung Ihrer Daten in ein Drittland können Sie jederzeit mit Wirkung für die Zukunft widersprechen.

6. Direkt mit der Studiendurchführung und der Nachsorge befasste Einrichtungen

Register DRKS	Deutsches Register Klinischer Studien<br>Bundesinstitut für Arzneimittel und Medizinprodukte, Dienstsitz Köln<br>Waisenhausgasse 36-38a, D-50676 Köln<br>Tel.: 0228 99 307 4942<br>E-Mail: poststelle@bfarm.de
Verantwortlicher für die Datenerhebung	Arzt im Studienzentrum, s. Deckblatt
Verantwortlicher für die studienbedingte Erhebung personenbezogener Daten und Datenverarbeitung	Prof. Dr. med. Erdem Güresir<br>Universitätsklinik für Neurochirurgie<br>Venusberg-Campus 1, D-53127 Bonn<br>Telefon: +49 228 287 11350<br>E-Mail: PICASSO@ukbonn.de
Datenschutzbeauftragte/r der Studienleitung/ lokaler Datenschutzbeauftragter:	Achim Flender, Datenschutzbeauftragter des Universitätsklinikum Bonn<br>Venusberg-Campus 1, Geb. 01, R 212a<br>D-53127 Bonn<br>Tel.: 0228-287-16075 oder 0228-287-14574<br>E-Mail: datenschutz@ukbonn.de
Datenschutz-Aufsichtsbehörde der Studienleitung	Landesbeauftragte für Datenschutz und Informationsfreiheit Nordrhein-Westfalen<br>Kavalleriestr. 2-4, D-40213 Düsseldorf<br>Tel: 0211-384240<br>E-Mail: poststelle@ldi.nrw.de
Datenschutz-Aufsichtsbehörde	Die Kontaktdaten der für das Bundesland Ihres Zentrums zuständigen Datenschutzbeauftragten finden Sie unter<br>https://www.bfdi.bund.de/DE/Infothek/Anschriften_Links/anschriften_links-node.html.

7. Ich bin damit einverstanden **ja ☐/ nein ☐ (bitte ankreuzen)**, dass mein Hausarzt

....

Name

über meine Teilnahme an der klinischen Studie informiert wird.

Ich willige in die Verarbeitung der genannten Daten ein.

Ich erkläre mich nachträglich bereit, an der oben genannten klinischen Studie freiwillig teilzunehmen.

Ein Exemplar der Patienten-Information und -Einwilligung sowie der Versicherungsbedingungen und -bestätigung habe ich erhalten. Ein Exemplar verbleibt im Studienzentrum.

....

Name des Patienten in Druckbuchstaben

....

Datum Unterschrift des **Patienten**

Ich habe das Aufklärungsgespräch geführt und die nachträgliche Einwilligung des Patienten eingeholt.

....

Name des Studienarztes / der Studienärztin in Druckbuchstaben

....

Datum Unterschrift des aufklärenden **Studienarztes / der Studienärztin**
