# Supplementary material for: PrImary decompressive Craniectomy in AneurySmal Subarachnoid hemOrrhage (PICASSO) trial: study protocol for a randomized controlled trial
Source: Trials. 2022 Dec 20;23:1027. doi: 10.1186/s13063-022-06969-4 (PMC9764529; doi:10.1186/s13063-022-06969-4)
Supplement: Supplementary file 1 — Additional file 1. Model consent form given to participants and authorized surrogates. [file 13063_2022_6969_MOESM1_ESM.zip › renamed_7367bR1.pdf]

Schriftliche Bestätigung
durch einen nicht an der klinischen Studie beteiligten Arzt

PICASSO

**Studientitel:**	**Primäre dekompressive Kraniektomie bei aneurysmatischer Subarachnoidalblutung**
**Studienkurztitel:**	**PICASSO**
**Prüfplan Code:**	**NCH_201702_PICASSO**
**DRKS-ID.:**	**DRKS00017650**

Studienzentrum und Studienarzt:
Prof. Erdem Güresir
Universitätsklinikum Bonn
Klinik und Poliklinik für Neurochirurgie
Gebäude 81
Venusberg-Campus 1
53127 Bonn

Feststellung einer Notfallsituation

für die Einbeziehung eines nicht einwilligungsfähigen Patienten in die oben genannte klinische Studie

durch den unabhängigen Konsiliararzt

Name: ....

Klinikadresse: ....

....

Telefon: ....

Frau /Herr

....
Name des Patienten

....
Geburtsdatum (TT/MM/JJJJ)

Leidet an

☐ aneurysmatischer Subarachnoidalblutung (aneurysmal subarachnoid hemorrhage (aSAH))

☐ ....

Um das Leben des Kranken zu retten, seine Gesundheit wiederherzustellen oder sein Leiden zu erleichtern

☐ ist eine Behandlung ohne Aufschub erforderlich.

☐ erscheint ein Behandlungsaufschub von maximal .... Stunden ohne Risiko für die Gesundheit des Patienten als vertretbar.

Der Patient wurde von mir darauf untersucht, ob er in der Lage ist, Wesen, Bedeutung und Tragweite seines Handelns sowie auch nur der klinischen Studie zu erkennen und seinen Willen hiernach zu bestimmen. Die Untersuchung hat ergeben, dass der Patient

☐ nicht einwilligungsfähig ist.

☐ einwilligungsfähig ist.

Die Einbeziehung in die oben genannte klinische Studie stellt dem Patienten in seiner aktuellen Situation

☐ einen persönlichen Behandlungsvorteil in Aussicht.

☐ keinen persönlichen Behandlungsvorteil in Aussicht.

Eine Erklärung über die Einwilligung konnte nicht herbeigeführt werden. Insbesondere konnte ein Betreuer für die Gesundheitssorge des Patienten nicht befragt bzw. nicht rechtzeitig bestellt werden. Unter Berücksichtigung sowohl der zu erwartenden Vorteile wie der denkbaren Risiken halte ich die Teilnahme des Patienten an der klinischen Studie

☐ für gerechtfertigt.

☐ nicht für gerechtfertigt.

Feststellung des mutmaßlichen Patientenwillens

Der mutmaßliche Patientenwille ist positiv in Bezug auf die Studienteilnahme

☐ Falls ja, der/die nächste/n Angehörige/n (Name und Verhältnis zum Patienten)

....

....

☐ Falls nein, kann der Patient <u>nicht an der Studie teilnehmen</u>.

☐ Falls unbekannt beschreiben Sie alle Bemühungen zur Ermittlung des mutmaßlichen Willens des Patienten und warum angenommen wird, dass der mutmaßliche Wille zur Studie positiv beurteilt wird

....

....

....

Einschlusskriterien:

In diese Studie eingeschlossen werden nur Patienten, die folgende Kriterien erfüllen:

- Männliche oder weibliche Personen, Alter ≥ 18 Jahre und ≤ 80 Jahre alt.
- Aneurysmatische Subarachnoidalblutung (SAH)
- WFNS (Grading-System zur Klassifizierung des Schweregrads einer Subarachnoidalblutung basierend auf dem klinischen Anfangszustand des Patienten) Grad 4 oder 5 bei der Aufnahme.
- Die Operation kann innerhalb der ersten 24 Stunden nach Beginn der Blutungssymptome durchgeführt werden.
- Eine frühzeitige Aneurysmabehandlung muss als möglich erachtet werden, um bei Patienten, die in Arm A randomisiert werden, die primäre dekompressive Kraniektomie innerhalb von 24 Stunden nach Beginn der Blutungssymptome durchführen zu können.

Ausschlusskriterien:

Patienten, auf die eines der folgenden Kriterien zutrifft, werden nicht in die Studie eingeschlossen:

- Moribunder Patient (vor Subarachnoidalblutung) aufgrund einer anderen Erkrankung
- SAH aufgrund einer anderen Ursache als einer Aneurysma-Ruptur (traumatische, arteriovenöse Malformation (AVM), Fistel, Dissektion)
- Patienten die das Follow-Up voraussichtlich nicht adäquat durchführen können
- Jede Vorerkrankung, die nach Einschätzung des Studienarztes ein Risiko für den Patienten darstellt, wenn eine Studientherapie eingeleitet wird oder die Teilnahme des Patienten an der Studie gefährdet.
- Patienten mit offensichtlichen Beweisen für irreparable Hirnstamm- oder thalamische Verletzung

Ich habe

- ☐ die Notfallsituation festgestellt
- ☐ die oben erwähnten Ein- und Ausschlusskriterien zur Kenntnis genommen
- ☐ den mutmaßlichen Patientenwillen überprüft und
- ☐ befürworte nach verantwortungsvoller und objektiver Risikoabwägung, die Studienteilnahme zum Wohl des Patienten.
- ☐ Ich bestätige, dass die Interessen des Studienteilnehmers gewahrt werden und seine medizinische Betreuung sichergestellt ist.

....

Name des/ der **nicht an Studie beteiligten Arztes/ Ärztin** in Druckbuchstaben

....

Ort, Datum | Unterschrift des/ der **nicht an Studie beteiligten Arztes/ Ärztin**

....

Name des Studienarztes / der Studienärztin in Druckbuchstaben

....

Ort, Datum | Unterschrift des **Studienarztes / der Studienärztin**
